# The impact of Livestock Manure Control Policy on human leptospirosis in Republic of Korea using interrupted time series analysis

**DOI:** 10.1017/S0950268817000218

**Published:** 2017-02-20

**Authors:** S. RYU, C. L. LAU, B. C. CHUN

**Affiliations:** 1 Division of Infectious Disease Control, Gyeonggi Provincial Government, Suwon, Republic of Korea; 2 Department of Epidemiology and Medical Informatics, School of Public Health, Korea University, Seoul, Republic of Korea; 3 Department of Global Health, Research School of Population Health, College of Medicine, Biology and Environment, Australian National University, Canberra, Australia

**Keywords:** Interrupted time series, leptospirosis, Livestock Manure Control Policy

## Abstract

Leptospirosis is a zoonotic disease that the pathogen can be transmitted to humans through the excretions of infected animals. In the Republic of Korea, the Livestock Manure Control Act was enforced in September 2007 to improve underground water hygiene. The objective of this study was to evaluate the impact of Livestock Manure Control Policy on the incidence and the trend of human leptospirosis. An interrupted time series analysis using the monthly incidence of leptospirosis was conducted based on data derived from the Korean National Surveillance System between January 1999 and January 2015. We used a Spearman correlation method to compare the level of leptospirosis incidence decrease between the metropolitan cities and rural provinces. The annual incidence of leptospirosis in South Korea decreased by 33% after policy enforcement of the policy. A significant change in the slope of human leptospirosis cases was observed after the policy enforcement (*β* = −0·09, *P* < 0·001). Moreover, we detected a clear association between the size of the rice paddy fields and the decrease in leptospirosis incidence in provinces (*r* = 0·817, *P* = 0·01). This study shows that the Livestock Manure Control Policy had significantly reduced human leptospirosis incidence in the Republic of Korea, in particular, in rural regions.

## INTRODUCTION

Leptospirosis is a zoonotic disease commonly reported in tropical and sub-tropical countries [1, 2]. Pathogenic leptospires typically inhabit the proximal renal tubules of their carriers’ (rodents, cattle, and pigs) kidneys. Infection in humans results from direct contact with the urine of infected animals or indirect contact with a contaminated environment through the mucous membranes or broken skin of the reservoir host animals, and can cause severe illness such as Weil's disease [3, 4].

Previous studies have shown that the incidence of leptospirosis follows distinct seasonal patterns and is strongly associated with high rainfall amounts and flooding, when leptospires are widely dispersed in the environment [5, 6]. Occupational exposure is also a significant risk factor in many countries and is particularly associated with agriculture and animal production (68% of total reported cases in South Korea, and 56·8% in Malaysia) [7–11]. In the Republic of Korea, rodents have been shown to be one of the major carriers of leptospirosis around swine farms; a survey in rodents conducted in 2008 found that 63·7% of rodents were infected [12, 13]. Moreover, infected cows and pigs can excrete hundred liters of infected urine per day and create highly contaminated environments on farms, where humans and other animal species could become infected. Thus, proper management of animal waste on farms is critical for the effective control of leptospirosis [14].

In September 2007, the Korean Ministry of Environment enforced the Livestock Manure Control Act, which makes it compulsory for livestock farmers to be equipped with appropriate sludge process facilities on their farms [15]. Since the main goal of this policy was to improve underground water sanitation, its effect on human leptospirosis incidence has not been studied.

In this study, we aimed to evaluate the effect of the Livestock Manure Control Policy on the incidence of human leptospirosis in the Republic of Korea.

## METHODS

### Study design

We conducted a retrospective interrupted time series analysis using national surveillance data. Since the Livestock Manure Control Act came into effect in September 2007, data were divided into two sets: those from January 1999 to September 2007 (before policy roll out), and those from October 2007 to December 2014 (after policy roll out) [16]. For further analysis of the policy effect on leptospirosis incidence decrease by administrative areas (the Republic of Korea has seven metropolitan cities and nine provinces), a correlation analysis was performed between the mean area of the rice paddy fields in the provinces and metropolitan cities and leptospirosis incidence. Possible confounders, including exposure to rodents and the amount of rainfall, were evaluated to measure differences between the pre- and post-roll out periods. The monthly incidence rates of Hemorrhagic Fever with Renal Syndrome (HFRS), transmitted by rodents, were used as a proxy measure of exposure to rodents [13].

### Data source

Monthly data of leptospirosis and HFRS incidence were collected from the Database of National Notifiable Infectious Diseases of the Korea Centers for Disease Control and Prevention between 1999 and 2015. The criteria mandating a notification for leptospirosis included clinically and epidemiologically suspicious cases, as well as laboratory-confirmed cases. Suspicious cases were defined as patients with the following criteria: (1) experiencing influenza-like illness with jaundice and/or renal failure, and/or hemorrhages, or (2) having occupations that put them at risk for direct contact with potentially infected animals (these included farm workers, sewage processors, veterinarians, and abattoir workers) [17, 18]. Laboratory-confirmed cases of leptospirosis were defined as those with positive microscopic agglutination tests or detection of the pathogen by blood culture, PCR (polymerase chain reaction) or antibody [18, 19].

Environmental data, including the total areas (hectare) of rice paddy field of provinces and metropolitan cities were derived from the Institution of Korean National Statistics [20]. The mean rice paddy field size was calculated during the study period.

### Statistical analysis

We calculated the annual incidence of leptospirosis and compared data from before and after the enforcement of the Livestock Manure Control Act using segmented regression analysis of time series data [16]. We considered the change of trend and level of the series. The following model was used for the analysis:

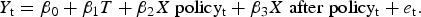

*Y*_t_ is the independent outcome variable of human leptospirosis incidence. *T* is the number of months starting in January 1999. *β*_0_ estimates the baseline level of the outcome at the beginning of the time series. *β*_1_ estimates the linear trend of the pre-intervention period where *T* is a continuous variable indicating the time in months at time t from the initiation of the study period. *β*_2_ estimates the changes in incidence where policy_t_ = 0 is before policy intervention and policy_t_ = 1 is after the intervention. *β*_3_ estimates the mean monthly trend in post-intervention period, where time after policy enforcement is a continuous variable indicating the number of months after implementation at time *t* and is coded as zero before the policy intervention. *e*_t_ is the random error at time *t*.

We utilized a Spearman correlation method to evaluate the association the mean areas of rice paddy field and the leptospirosis incidence at the provincial level. To evaluate differences in possible confounders (amount of rainfall and exposure to rodents) between before and after the policy roll out, the Mann–Whitney *U* test and a Poisson regression analysis were used. The statistical package R version 3.2·4 (R Foundation for Statistical Computing, Vienna, Austria) was used for all analyses.

## RESULTS

### Trend and association between leptospirosis incidence and implementation of the Livestock Manure Control Act

**Fig. 1.**
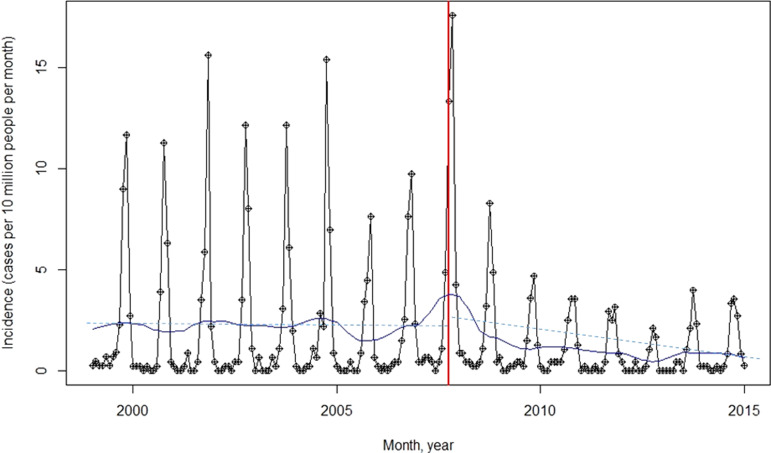
The incidence and the trend of leptospirosis. The figure shows the human leptospirosis incidence (solid line with circle) and the trend (solid line without circle). The vertical line indicates September 2007, when the Livestock Manure Control Act enforced (solid vertical line). The dashed line indicates the trend of before enforcement of policy (slope: −0·01, *P* = 0·69), and post-enforcement of policy (slope: −0·02, *P* < 0·01).

The annual incidence of leptospirosis was 29·14 per 10 million in 1999, peaking at 43·99 in 2007, followed by a decrease to 12·09 per 10 million in 2014 (Fig. 1). We found a significant association between the enforcement of the Livestock Manure Control Policy with a 33% decrease in leptospirosis incidence during the post-enforcement period (95% confidence interval (CI) 13–53, *P* < 0·01). The interrupted time series analysis showed that the leptospirosis incidence was 2·23 per 10 million at the beginning of study period (January 1999). No significant changes before and immediately after the policy roll out were observed. However, a significant decrease in leptospirosis incidence (0·02 per 10 million per month) after policy enforcement (Table 1).

**Table 1.** Interrupted time series regression analysis of leptospirosis

	Coefficient	Standard error	*t* value	*P* value	*R*^2^
Intercept *β*_0_	2·23	0·09	25·510	<0·01	0·64
Baseline trend *β*_1_	−0·01	0·02	−0·39	0·694	
Level change *β*_2_	0·05	0·13	0·346	0·729	
Trend change *β*_3_	−0·02	0·01	−9·09	<0·01	

### Level of leptospirosis incidence decrease in the metropolitan cities and provinces

**Fig. 2.**
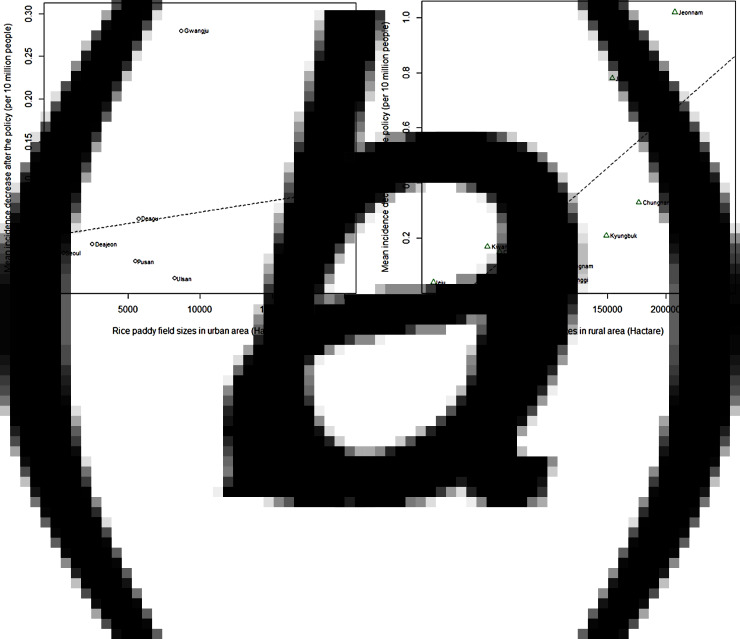
Association between rice paddy field areas and the decrease in human leptospirosis incidence in metropolitan cities and provinces. This figure shows the decrease in leptospirosis in urbanized metropolitan cities (*a*) and provinces (*b*). The level of decrease on leptospirosis incidence in metropolitan cities is relatively smaller with no significant association with rice paddy field areas (*r* = 0·11, *P* = 0·82). The level of decrease on leptospirosis incidence in provinces is bigger than metropolitan cities and has a significant association with the rice paddy field areas (*r* = 0·82, *P* < 0·01).

The mean decrease in leptospirosis incidence after policy enforcement were 0·06 per 10 million people in seven metropolitan cities (with a mean rice paddy field area of 6716 hectare) and 0·32 per 10 million people in the nine provinces (with a mean rice paddy field area of 1 12 414 hectare). Although we found no significant association between the effect of the policy on leptospirosis incidence and the area of rice paddy fields in metropolitan cities, a statistically significant association was observed for the nine provinces (*r* = 0·82, *P* = 0·01; Fig. 2).

### Environmental drivers of leptospirosis

**Fig. 3.**
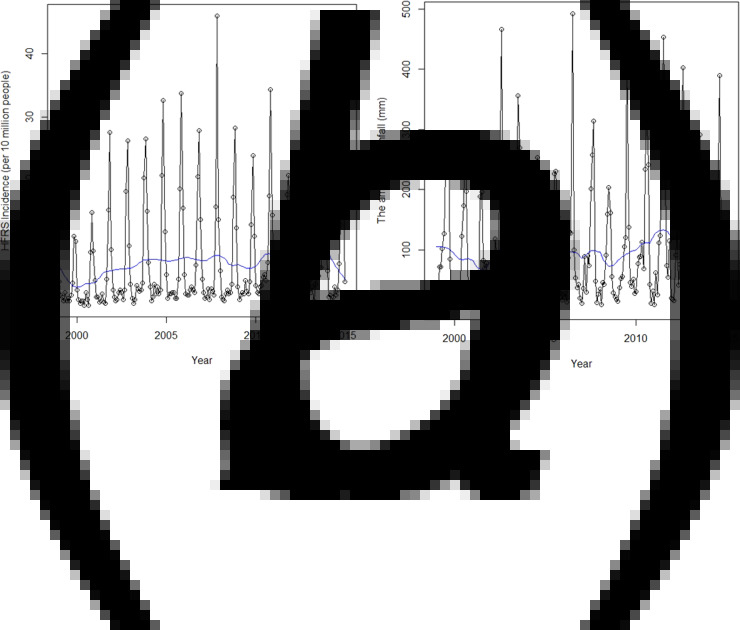
The environmental drivers of leptospirosis. (*a*) The incidence (solid line with circle) and trend of HFRS (a proxy of exposure to rodents; solid line without circle). There was no significant change with HFRS during study period (RR = 1·15, *P* = 0·06). (*b*) The amount of rainfall (solid line with circle) and its trend (solid line without circle). There was no significant differences between pre- and post-period of Livestock Manure Control Act enforcement (*P* = 0·97).

We did not find a significant association between policy enforcement and HFRS incidence, a proxy measure of exposure to rodent (relative ratio (RR) 1·15, 95% CI 1·07–1·15, *P* = 0·06). In addition, no significant differences were seen in the amount of rainfall between pre- and post-enforcement periods (Fig. 3).

## DISCUSSION

Our study has shown that enforcement of the Livestock Manure Control Act in the Republic of Korea was associated with a decrease in human leptospirosis incidence. We found strong positive correlation between the decrease in leptospirosis incidence and the mean area of rice paddy fields in the provinces. No significant change was found in environmental factors such as the amount of rainfall and exposure to rodents.

In 1990s, South Korean government promoted the use of organic waste including livestock manure to curb the excessive use of chemical fertilizers that had caused an imbalanced of nutrients in the soil [21]. Previous studies have however shown that rice paddy fields and water streams can be contaminated by discharging livestock waste, which is a possible vector for spreading leptospirosis [21–23]. Thus, our findings support the notion that the enforcement of a Livestock Manure Control Act can reduce human leptospirosis incidence.

Moreover, several studies have shown that there is a positive association between water logged land, which provides a favorable environment to sustain leptospires, and leptospirosis incidence [5, 6]. This is consistent with our study that a positive association between rice paddy field size and the leptospirosis incidence was found (*r* = 0·94, *P* < 0·01). Thus, this supports our finding that the decrease in human leptospirosis incidence after the policy enforcement was larger in the provinces, which had larger rice paddy field sizes.

This study has several limitations. First, the Database of National Notifiable Infectious Diseases includes clinically and epidemiologically defined cases that have not been laboratory confirmed. However, these data provide the best proxy measure of the leptospirosis cases through a nationally trusted institution. Second, we used HFRS data as a proxy for exposure to rodent. A proxy measure is not in itself directly relevant; yet, it can be used for statistical analysis in place of immeasurable variables. Several studies have shown that HFRS and rodent density exhibit a significant correlation as HFRS is caused by Hantaan or Seoul viruses, of which mice are the main carriers [13, 24]. Third, our finding could have been affected by confounders due to the ecological nature of this study. Although major known confounders such as the amount of rainfall and exposure to rodents were considered in our study, other unknown confounders could have affected leptospirosis incidence during the post-enforcement period.

This is the first study to quantify the effect of a Livestock Manure Control Policy on the incidence of zoonotic disease in humans using standardized national data spanning 15 years. Furthermore, our data identified a statistically significant association between the policy's effect and the mean rice paddy field size on the provincial level.

## CONCLUSION

Our study found a significant decrease in the human leptospirosis incidence after the enforcement of the National Livestock Manure Control Act in South Korea, in particular in the nine provinces. Our study suggests that livestock manure control could be an important strategy for the leptospirosis control and prevention.
